# PRAGAN: Progressive Recurrent Attention GAN with Pretrained ViT Discriminator for Single-Image Deraining

**DOI:** 10.3390/s22249587

**Published:** 2022-12-07

**Authors:** Bingcai Wei, Di Wang, Zhuang Wang, Liye Zhang

**Affiliations:** College of Computer Science and Technology, Shandong University of Technology, Zibo 255000, China

**Keywords:** deep learning, image deraining, neural network, vision transformer, generative adversarial network

## Abstract

Images captured in bad weather are not conducive to visual tasks. Rain streaks in rainy images will significantly affect the regular operation of imaging equipment; to solve this problem, using multiple neural networks is a trend. The ingenious integration of network structures allows for full use of the powerful representation and fitting abilities of deep learning to complete low-level visual tasks. In this study, we propose a generative adversarial network (GAN) with multiple attention mechanisms for image rain removal tasks. Firstly, to the best of our knowledge, we propose a pretrained vision transformer (ViT) as the discriminator in GAN for single-image rain removal for the first time. Secondly, we propose a neural network training method that can use a small amount of data for training while maintaining promising results and reliable visual quality. A large number of experiments prove the correctness and effectiveness of our method. Our proposed method achieves better results on synthetic and real image datasets than multiple state-of-the-art methods, even when using less training data.

## 1. Introduction

Rain patterns in an image will affect the visibility of the image and cause considerable trouble to imaging instruments. Degradation phenomena, such as rain streaks and fog, will greatly decrease the accuracy of visual tasks, especially for high-level tasks. Therefore, removing rain from rainy images has become classical in down-stream visual tasks, while, single-image deraining is a challenging task in low-level visual research fields.

Deep learning, relying on its strong representation and mapping fitting ability, has made great achievements in the field of computer vision in recent years. Not only in high-level visual tasks, such as image classification [1], object detection [2], semantic segmentation [3], and person reidentification [4], has deep learning occupied a dominant achievement, but also in the low-level visual tasks. For visual representations, the depth of network is very important [5], but simply deepening the neural network will make it difficult to train. Since ResNet [6] solved this problem, the application of convolutional neural network (CNN) in computer vision has shown a spurt of development [7,8]. Later researchers mimicked human visual attention by adding attention mechanisms [9,10] to CNN, allowing it to allocate more computing resources to parts that contain significant information based on dynamic weight scores [11]. Recently, with the excellent performance of self-attention [12], ViT [13] has re-examined the choices of network backbone. Meanwhile, CNN can also be combined with GAN and recurrent neural network (RNN), respectively. Using the powerful generation ability of GAN and the outstanding temporal modeling capability of RNN, attractive achievements have been made in image generation [14] and deblurring [15], video super-resolution [16], and denoising [17] tasks.

Single-image deraining is a hot issue because the images captured in rainy days will be significantly degraded by rain patterns, so computer vision tasks are difficult to perform. In contrast to the model-based or prior-based methods in traditional algorithms, learning-based methods are applied to image rain removal, and can achieve more promising results with better generalization ability, while requiring no prior knowledge. In detail, combined with image processing domain knowledge, Fu et al. [18] proposed a modestly sized CNN to modify the objective function for image deraining. Yang et al. [19] created a recurrent rain detection and removal network that could jointly detect and remove rain from single images. Zhang et al. [20] proposed a density-aware, multi-stream, densely connected network for joint rain density estimation and deraining that can automatically determine the rain-density information. As for single-image deraining, Zhang et al. [21] proposed ID-CGAN (image deraining conditional generative adversarial network) by leveraging the powerful generative modeling capabilities of conditional GAN. Ren et al. [22] proposed a progressive recurrent network that can take the advantage of recursive computation while exploiting the dependencies of deep features across stages. Attention mechanisms, such as CNN, GAN, RNN, and ViT, are all excellent components of deep learning, which can be used as components to design a network that combines the advantages and characteristics of a variety of structures. The use of these network structures alone cannot obtain a satisfactory effect, therefore, our motivation was to give full play to the advantages of various network structures by integrating and collocating multiple network structures. On the other hand, training of complex neural networks requires a lot of data, which means it takes a lot of time simultaneously. Therefore, the efficient use of data will make training easier. Given that generators are the more important member, there have been few studies on discriminators and the stability of their training. In order to solve the above problems, in this study, we propose a progressive recurrent attention generation adversarial network, the generator for which includes a convolutional block attention module [10] (CBAM) and convolutional LSTM [23] (ConvLSTM). At the same time, a pretrained ViT is proposed as a discriminator to organize the adversarial training with the generator. Finally, we introduce a training method that can use only a portion of training image pairs while obtaining results beyond the amount of data. Detailed ablation experiments and comparative experiments have proven the rationality and effectiveness of our proposed method.

The main contributions of this work are as follows:

1. We propose an adversarial model using a pretrained ViT discriminator. We utilize ViT’s powerful fitting ability in computer vision while minimizing its drawback of requiring large amounts of data for pretraining. To our best knowledge, there has been little work to improve the performance of discriminators in image deraining, and we are the first to propose a pretrained ViT discriminator to improve the overall performance of GAN.
2. We propose a data reselection algorithm, called DRA. To be specific, the training data are reselected at a specific time in the process of network training. Compared with the fixed part of training data, the rain removal effect of our model can be significantly improved by using this algorithm.
3. A large number of comparative experiments and ablation experiments on synthetic and real datasets prove the effectiveness and rationality of our proposed method.

### 1.1. Single-Image Deraining

Compared with video deraining tasks, which that can use inter-frame temporal information, significantly less information can be fully utilized in individual images for single-image deraining. Therefore, it is obviously more difficult and challenging to remove rain streaks in single images. In early studies, the rain model is usually simply expressed as Formula (1):

(1) $$O=B+\tilde{S}$$

where O is the input image with rain streaks, B is the background image, and $\tilde{S}$ is the rain streak layer. Yang et al. [6] proposed a new model in order to realistically simulate the rain streak phenomena in the real world. By accommodating streak accumulation and overlapping rain streaks with different directions, this model can both comprise of multiple layers of rain streaks and represent diversity of rain streaks. The new rain model is expressed as Formula (2):

(2) $$O=\alpha (B+\sum_{t=1}^{s}{\tilde{S}}_{t}R)+(1-\alpha )A$$

where ${\tilde{S}}_{t}$ is the rain streak layer in the same direction, which has effects of atmospheric shading; S is the maximum number of rain streak layers; and t is the index of these layers. R represents binary values of 0 or 1, 0 representing areas without rain and 1 representing areas with rain. α represents the atmospheric propagation transmittance that is common in image dehazing and A represents the global atmospheric light value.

### 1.2. ConvLSTM and GAN

To solve the problem that storing information over extended time intervals is time-consuming, Sepp et al. [24] proposed long short-term memory (LSTM). As a recurrent version of the cascade correlation learning architecture, recurrent cascade correlation can learn from examples to map an input sequence to the desired output sequence while preserving the benefits of cascade correlation, such as fast learning. LSTM can lead to more successful runs than recurrent cascade correlation while learning much faster. However, the fully connected LSTM (FC-LSTM) cannot encode spatial information in handling spatiotemporal data. To overcome this major drawback of LSTM, Shi et al. [23] proposed ConvLSTM, which is more suitable for spatiotemporal data than FC-LSTM while preserving the advantages of it. ConvLSTM consists of an input gate $i_{t}$, an output gate *o_t_*, a forget gate $f_{t}$, and a memory cell $C_{t}$ [25]. The key equations of ConvLSTM are shown in Formula (3):

(3) $$\begin{array}{l} i_{t}=\sigma (W_{xi}*X_{t}+W_{hi}*{ℋ}_{t-1}+W_{ci}\circ C_{t-1}+b_{i})\\ f_{t}=\sigma (W_{xf}*X_{t}+W_{hf}*{ℋ}_{t-1}+W_{cf}\circ C_{t-1}+b_{f})\\ C_{t}=f_{t}\circ C_{t-1}+i_{t}\circ tanh(W_{xc}*X_{t}+W_{hc}*{ℋ}_{t-1}+b_{c})\\ o_{t}=\sigma (W_{xo}*X_{t}+W_{ho}*{ℋ}_{t-1}+W_{co}\circ C_{t}+b_{o})\\ {ℋ}_{t}=o_{t}\circ tanh(C_{t}) \end{array}$$

where ∘ and ∗ denote Hadamard product and convolution operator. $X_{t},H_{t},W_{*},b_{*}$ are input tensor, hidden state tensor, network weights, and bias terms, respectively.

By simultaneously training a generative model G and a discriminative model D via an adversarial process, GAN can represent even degenerate distributions with no approximate inference better than methods based on Markov chains [26]. The training objective of D is to distinguish between data generated by *G* and real data as much as possible. The training goal of G is to make D unable to distinguish between them. The adversarial process is shown as a two-player minimax game in Formula (4):

(4) $$\underset{G}{\min}\;\underset{D}{\max}V(D,G)=E_{x∼P_{data(x)}}[\log D(x)]+E_{z∼P_{z(x)}}[\log(1-D(G(z)))]$$

where $P_{data(x)}$ and $P_{z(x)}$ are the distributions of real data and generated data, meanwhile, D(x) and D(G(z)) are the probabilities of the discriminator judging real or generated data as true, respectively. GAN has a disadvantage that D must be synchronized well with G during training [26] while suffering from training instability [27]. Therefore, the structures of G and D must be well-designed, and the components used in the proposed network will be described in the following section.

### 1.3. CBAM and ViT

**Figure 1:**
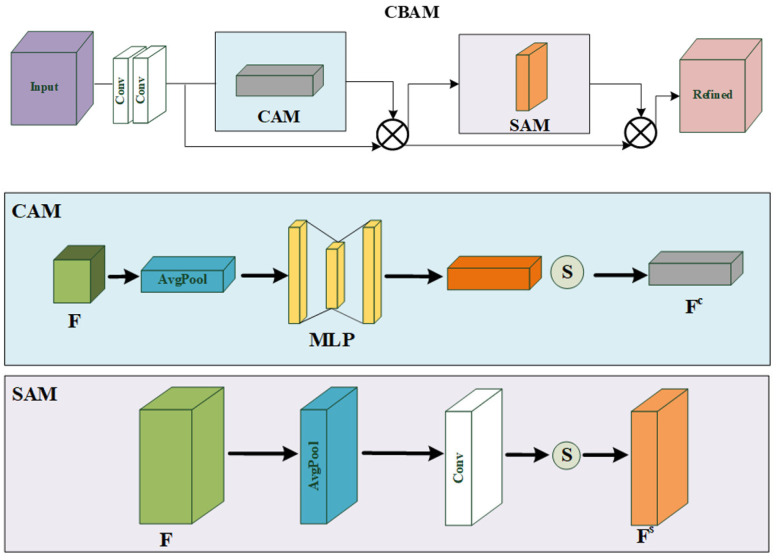
Detailed structure of CBAM.

Hu et al. [12] proposed the SE module, which uses global average-pooled features to compute channel-wise attention for exploiting the inter-channel relationship. However, the SE module is suboptimal because it only focuses on the channel dimension. CBAM [10] can sequentially infer attention maps along not only the channel but also the spatial dimension to get better inter-dependencies than [9]. The overall attention process of CBAM [10] is shown in Formulas (5) and (6):

(5) $$\begin{array}{l} F^{\prime }=M_{c}(F)⊗F\\ F^{\prime \prime }=M_{s}(F^{\prime })⊗F^{\prime } \end{array}$$

where $F^{\prime }$ and $F^{\prime \prime }$ are the intermediate feature map and the final refined output, while ⊗ denotes element-wise multiplication, in which:

(6) $$\begin{array}{l} M_{c}(F)=\sigma (W_{1}(W_{0}(F_{avg}^{c}))+W_{1}(W_{0}(F_{\max}^{c})))\\ M_{s}(F)=\sigma (f^{7\times 7}([F_{avg}^{s};F_{\max}^{s}])) \end{array}$$

where $F_{avg}^{*}$, $F_{\max}^{*}$, $W_{*}$, and $f^{7\times 7}$ denote average-pooled features, max-pooled features, CNN’s weights, and convolution operations with a 7 × 7 filter, respectively. Further, the structure of the CBAM is shown in Figure 1.

Based solely on self-attention mechanisms, transformer [12] is the de facto standard for natural language processing tasks. Applications of pure transformer [13] or its variants [28,29] to computer vision tasks prove the superiority of transformer over CNN and RNN. By flattening the loss landscapes [30], multi-head self-attentions (MSAs) in transformer improve not only accuracy but also generalization, which gives transformer excellent fitting and representation abilities. As a discriminator, we only used the transformer encoder, which includes a MSA module and a feed-forward network (FFN). The size of input $f_{pi}$ is the same as that of output patch in encoder $f_{E_{i}}\in {\mathbb{R}}^{P^{2}\times C}$, and the whole calculation of transformer can be formulated in Formula (7):

(7) $$\begin{array}{l} y_{0}=[E_{p1}+f_{p1},E_{p2}+f_{p2},\ldots ,E_{pn}+f_{pn}],\\ q_{i}=k_{i}=v_{i}=LN(y_{i-1}),\\ y_{i}^{\prime }=MSA(q_{i},k_{i},v_{i})+y_{i-1},\\ y_{i}=FFN(LN(y_{i}^{\prime }))+y_{i}^{\prime },i=1,\ldots ,l\\ {}[f_{E1},f_{E2},\ldots ,f_{E_{n}}]=y_{1} \end{array}$$

in which the self-attention in MSA can be unified as (8):

(8) $$Attention(Q,K,V)=soft\max(\frac{Q\cdot K^{T}}{\sqrt{d_{k}}})$$

where q,k,v are query, key, and value used in MSA; Q,K,V are vectors packed together into three different matrices which are derived from different inputs [31], respectively. In addition, l denotes the number of layers in the encoder and LN is the layer normalization [32] applied before every block.

## 2. Proposed Method

In the second chapter, we mainly introduce three parts. Firstly, we mention the overall network structure and progressive recurrent loss function. The second part introduces a confrontation model using a pretrained ViT discriminator. Finally, we introduce an effective training method: reselecting data progressively.

### 2.1. Network and Loss Function

**Figure 2:**
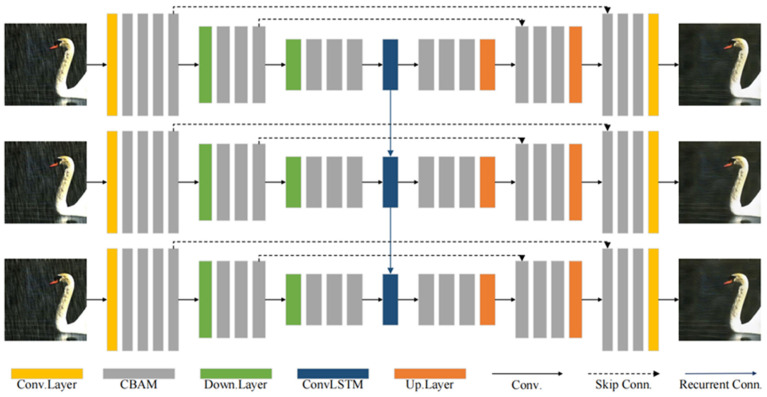
The overall structure of our proposed generator. The CBAM part is shown in Figure 1. Our generator is a variant of the recurrent neural network, which offers three cycles in the figure above. The parameters of the three cycles are shared, that is, only one-third of the parameters of the overall network. The generator does not need to be pretrained. In the adversarial training, the pretrained discriminator is used to conduct adversarial training with the generator proposed above.

We promote the guiding role of loss function [33] for end-to-end single-image rain removal. The whole structure of our generator is shown in Figure 2, the generator was inspired by the manner of progressively coarse-to-fine restoration from degraded to sharp images in [34,35,36], methods of sharing network parameters in [37], and RNN for deraining [22,36]. 

We applied one loss to each loop in the training process of the generator to achieve a progressive recurrent loss. Specifically, in the first loop, we used MSE loss, which is expressed in Equation (9):

(9) $${ℒ}_{mse}=\frac{1}{N}{\left\|B_{1}-B\right\|}_{2}^{2}$$

where $B_{1}$ is the output of first loop and N is the number of elements in $B_{1}$ to normalize. In the second loop, we employed the EDGE loss, which is expressed in Equation (10):

(10) $${ℒ}_{edge}=\sqrt{{\left(Lap(B)-Lap(B_{2})\right)}^{2}+\varepsilon^{2}}$$

where Lap(∗) denotes the edge maps extracted from images via Laplacian operator [38] and ε is set to 0.001. In the last loop, as the final result, we chose structural similarity (SSIM) [39] loss, which can take into account the overall coordination between predicted deraining images and labels. The SSIM between image *X* and image *Y* can be expressed as Formula (11):

(11) SSIM(X,Y)=l(X,Y)c(X,Y)s(X,Y)

where *l(X, Y), c(X, Y)*, and *s(X, Y)* are luminance component, contrast component, and structure component of SSIM, respectively. The SSIM loss between final output $B_{3}$ and label can be defined as Formula (12):

(12) $${ℒ}_{SSIM}=1-SSIM(B_{3},B)$$

**Figure 3:**
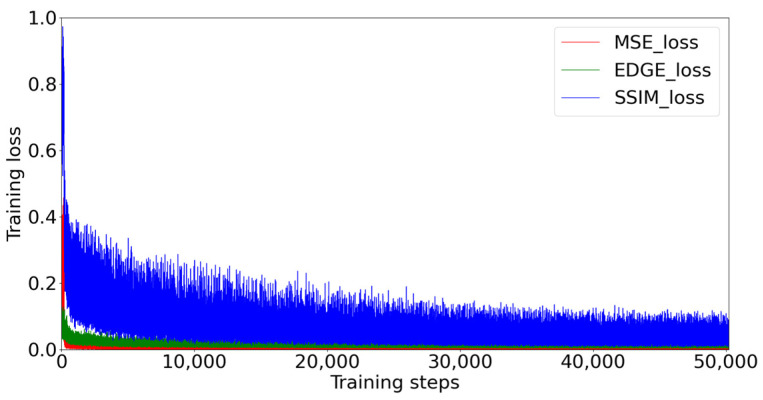
Numerical comparison of three losses during model training.

In order to avoid the burden of fine-tuning parameters [22], we conducted a prior analysis of the above three loss values, as shown in Figure 3. Therefore, we simply arranged the order by numerical value from small to large. Therefore, the final loss used for our model is defined as Formula (13):

(13) $$Loss_{all}={ℒ}_{mse}+{ℒ}_{edge}+{ℒ}_{SSIM}$$

### 2.2. Discriminator: Pretrained ViT

Due to their capacity for long-range representation [40] and faculty for flattening loss landscapes [30], transformer-based models show high performance for visual tasks with less need for vision-specific induction [31]. Multiple tasks [41,42,43] have revealed that transformer-based models heavily rely on massive datasets for large-scale training, which may be the key to achieving its inductive bias [13]. However, pretraining [44] on large-scale datasets (e.g., ImageNet [45]) is both very demanding on hardware and does not necessarily improve the final target task accuracy [46].

**Figure 4:**
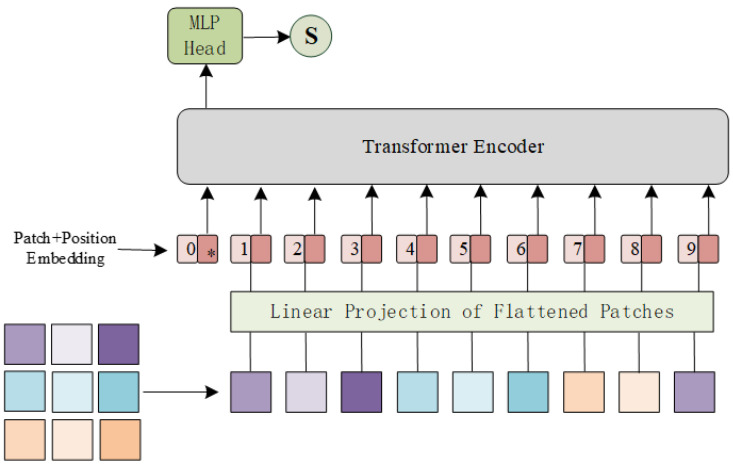
The detailed structure of the transformer discriminator used in this article. In addition, the ‘*’ symbol represents class token.

**Figure 5:**
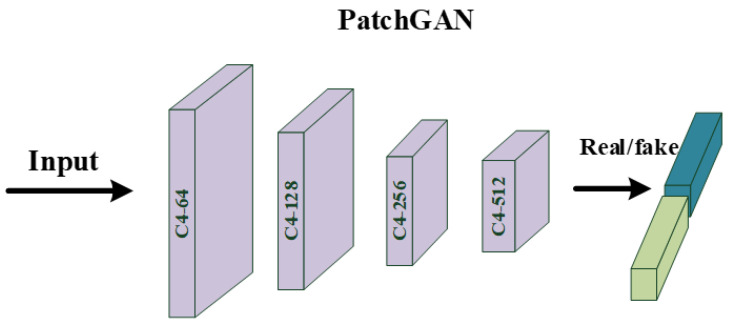
The detailed structure of the classical PatchGAN.

**Figure 6:**
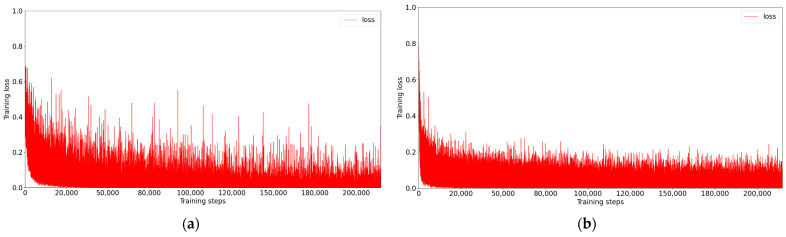
Comparison of losses convergence in pretraining. Note, our aim is not to make a performance comparison between these two, but rather to explore the wider use of ViT [13] and pretraining for generative tasks from the perspective of a GAN’s discriminator. (**a**) PatchGAN [47] on 128 × 128 patches. (**b**) ViT [13] on 128 × 128 patches.

**Figure 7:**
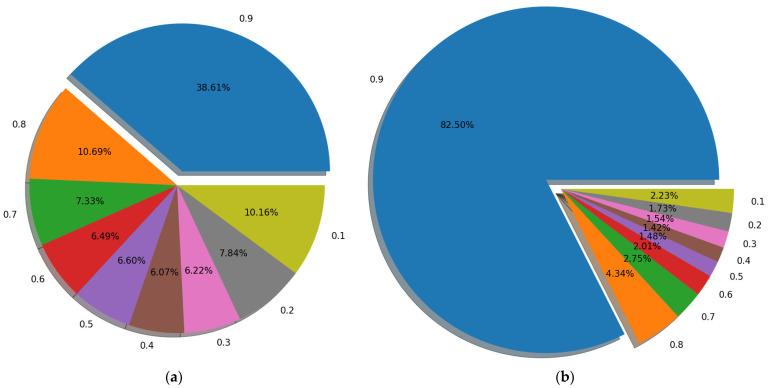
The difference between the predicted values of all image pairs after training. The goal of both networks is to return 1 to the image without rain and 0 to the image with rain. The number in the figure is the return value of the image without rain minus the return value of the image with rain. That is, the larger the difference is, the stronger the network’s discrimination ability is. As shown in the figure, on the image patches of 128 × 128, ViT [13] performs better than PatchGAN [47]. (**a**) PatchGAN [47] on 128 × 128 patches. (**b**) ViT [13] on 128 × 128 patches.

In this section, we give a detailed description of a proposed strategy that uses our pretrained ViT as the discriminator of GAN. Compared with the large-scale dataset that includes over tens of millions of images, we used less than 3 × $10^{4}$ images for training. Given that this pretraining process can be regarded as a binary classification task, the number of training iterations is small while the effect is good. To demonstrate the superiority of ViT over CNN, we also trained a classical CNN, called PatchGAN [47], which is often used as a discriminator in image restoration tasks [48,49]. The PatchGAN [47] network mainly includes: C64−C128−C256−C512. $C_{k}$ presents a 4 × 4 Convolution + BatchNorm + LeakyReLU block with stride two and k filters. The parameters of these LeakyReLU activation functions were set to 0.2 and the last two layers of this network are made up of a 4 × 4 convolution layer, for which stride and filter number were set to one, and an average pooling layer. Meanwhile, the ViT used as a discriminator has 16 patch sizes, 768 embedding dimensions, 6 MSAs Blocks, and 12 attention heads. The detailed structures of ViT [13] and PatchGAN [47] are shown in Figure 4 and Figure 5. By recording the loss function, as shown in Figure 6, ViT [13] converges faster and is more stable than CNN during training. Further, as shown in Figure 7, by testing the trained network on whole data, we found that, as a discriminator, pretraining ViT can better distinguish images with rain from clear images. After pretraining, this ViT has been fully equipped with the ability to distinguish whether the training data contain rain.

### 2.3. Reselecting Data Progressively: Train More Effectively

Nowadays, deep neural networks often require a large amount of data for training to converge. As described in the previous section, pretraining on large-scale datasets requires fairly good hardware conditions and very long time, but does not necessarily improve final target task accuracy [46]. Not only that, in order to comprehensively explore the competence, models for single image deraining also require massive data for training [36], which also increases the difficulty for this task to a certain extent.

To solve these problems, we propose an algorithm for progressively random reselection of data, which is inspired by the coarse-to-fine principle that has been proved to be effective [50] by other image restoration tasks [35,51]. Specifically, randomly select a portion from the entire training set at the beginning and then reselect it several times. By reselecting training data at the end of a specific training epoch, we can achieve better results than using the same amount of training data without reselecting. In addition, in accordance with the principle of coarse-to-fine, we interval different training epochs to reselect the data, which makes the intervals change from large to small. At the end stage of network training, the data are reselected every two epochs, while in the initial stage of network training, data are reselected every twenty-five epochs. From the perspective of network generalization performance, using different data for training every once in a while can simply inhibit over-fitting. At the same time, in contrast to the discriminator, our generator does not need pretraining, although pretraining will not automatically help reduce overfitting [46]. Each process is carried out before one training epoch; compared with the time required for training, time consumption of reselecting data can be ignored, but it can perform better results. The process of reselecting data is summarized in Algorithm 1:
**Algorithm 1:** Reselecting Data Progressively.**Parameters:** *M* = 251: total epoch number for training, *E* = 50: number of epochs included in one stage of reselecting data progressively, *D*: all the training data, *R* = 4: ratio of overall data selection, *List* = [25, 10, 5, 2]: a list of epoch values for reselecting data, *S* = [0, *List*[0]]: a list for saving the number of rounds for which data should be reselected, *L* = [0, …, len(D)]: a list of integers from 0 to the length of D, *Loader*: dataloader in Pytorch1. **for** *i* = 0 to *len(List) do*
2.       **while** *S*[−1] < (E*(*i* + 2)) **do** 3.             *S*.append(*List*[*i*] + *S*[−1]) 4.       **end while** 5. **end for** 6. **for** *i* = 0 to *M* **do** 7.       **if** *i* in *S* **then** 8.              Shuffle(*L*) 9.              *Part* = [*D*[*j*] for *j* in *L*[0:(len(*D*)//*R*)]] 10.             Loader(*Part*) 11.     **end if** 12.     Train one epoch 13. **end for**

## 3. Experimental Results

### 3.1. Implementation Details

We implemented our model with the pytorch library. The generator was able to be divided into three stages based on the size of feature map. After each down-sampling, the number of channels in the convolution layer was twice that before. The number of channels in the convolution layer at the beginning of the network was 32, and the convolution kernel size of all convolution layers was 3. The image patches used in all experiments were 256 × 256. Due to hardware limitations, specific ablation experiments may use different batch sizes. All the generators in different ablation experiments used Adam [52] optimizer for training, and the initial learning rate was 0.0002, which steadily decreased to 1 × 10^−6^ using the cosine annealing strategy [53]. In contrast to the generator, the initial learning rate of the discriminator during pretraining was 2 × 10^−5^, and AdamW [54] optimizer was used for optimization. Horizontal and vertical flips were randomly applied for data augmentation. In addition to pretraining the discriminator, our experiments were conducted on an NVIDIA RTX 3060 GPU. Further details may be found in [55].

On several synthetic datasets, our proposed PRAGAN was compared with seven state-of-the-art models, i.e., DerainNet [5], RESCAN [55], DIDMDN [7], UMRL [56], SEMI [57], PreNet [22] and MSPFN [36]. All other methods were configured as in [36], and we used the results provided by [36] to establish a re-evaluation of image quality by employing the peak signal to noise ratio (PSNR) and SSIM in scikit-image. All datasets used for training included Rain14000 [58], Rain1800 [6], Rain800 [21], and Rain12 [59], with a total of 13,712 image pairs, which we call MIX.

On real-world rainy image datasets, according to the configuration in [60], we only trained the proposed model on the Rain100L [6] training set, we call it Train200. Train200 has a total of 200 image pairs, and PRAGAN was tested on Internet-Data [57] and SPA-Data [61]. These two datasets contain 147 rainy images and 1000 image pairs, respectively. Given that Internet-Data [57] has no ground truth, we only provide visual comparison with several state-of-the-art models in Figure 7.

### 3.2. Ablation Studies

In this section, we provide the contributions of different designs quantitatively.

#### 3.2.1. Network Structure and LOSS

By removing CBAM and ConvLSTM, we verified the necessity of using them. For progressive recurrent loss, experiments have shown that this loss can achieve better results than adding three losses to one loop or using MSE loss to measure the prediction value of each loop directly. Finally, it should be noted that our network only inputs the original rain image each loop, rather than the predicted value of the previous loop. We found through experiments that for PRAGAN, doing so will bring performance losses. The training set and testing set used in all experiments in this section were Rain800 [21] and Test100 [21]; mini-batch size and training epoch were 1 and 101. All the results are shown in Table 1.

#### 3.2.2. Pretraining ViT as Discriminator

In this section, we compare the ViT pretrained on 128 × 128 and 256 × 256 image patches, as shown in Table 2 and Table 3. For the smaller image patches, we set batch size to 64, while for the larger image patches, due to the hardware limitation, we set batch size to 16. The number of training epochs and the initial learning rate were 502 and 2 × 10^−5^, respectively. When the discriminator was used for adversarial training, the initial learning rate was 1 × 10^−5^. Our discriminator used AdamW [54] as optimizer in pretraining and adversarial learning. During pretraining and adversarial training, the loss of both patch sizes for discriminator was BCEloss. The training dataset for pretraining was MIX. In order to better display the superiority of pretraining for ViT, as for the smaller patch, we trained the network on a quarter of the MIX training set. Meanwhile, for the larger patch, we used a quarter of Rain1800 [19] for training. Pretraining ViT can effectively help the generator to improve the performance of image deraining.

#### 3.2.3. Reselecting Data Algorithm

In this part, we studied the reselecting data algorithm with small and large amounts of training data to better demonstrate its effectiveness. Specifically, the smaller one was Rain800 [21] and the larger one was a quarter of MIX, including 700 and 3426 image pairs, respectively. Batch size of the former was 1, the latter was 2. The number of training epochs and the size of image patches were 251 and 256, respectively, and relevant results are shown in Table 4 and Table 5. With the increase of the amount of training data, the corresponding image evaluation index will also increase. Meanwhile, using same amount of data, by employing a reselecting data algorithm, the deraining task can obtain better results.

### 3.3. Comparison with Other Methods

#### 3.3.1. Synthetic Images

**Figure 8:**
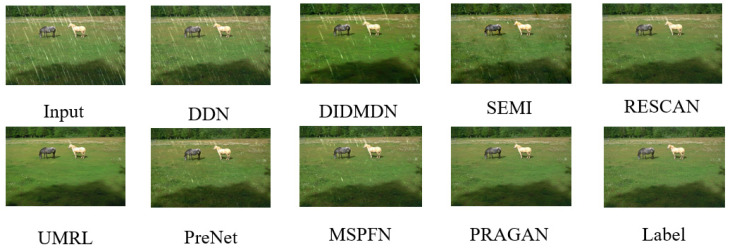
Deraining results from the Rain100L [19] testing set. Rain100L [19] consists of 100 image pairs for testing with one type of rain streak. It can be seen from the figure that most of the methods can remove rain streaks to a certain extent, but our PRAGAN can almost remove all the rain streaks compared with other methods, and restore images closer to ground truth.

**Figure 9:**
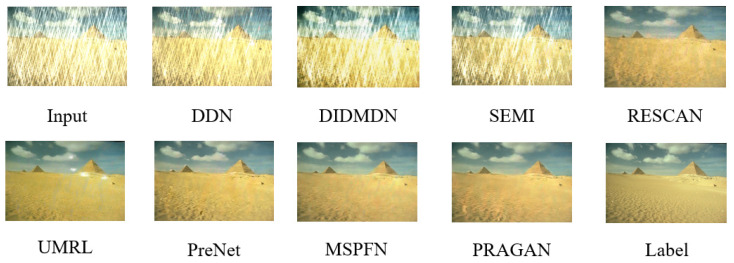
Deraining results from the Rain100H [19] testing set. In contrast to the relatively simple Rain100L [19], Rain100H [19] contains five types of streak directions, so part of the rain removal method was not effective. Our method needed only a quarter of the 13,712 image pairs for training.

Through training on one quarter of the MIX training set, combined with DRA and pretraining of the ViT discriminator, we obtained the best results with the proposed method. We compared it with eight state-of-the-art methods. Due to the relatively long time, we remeasured the image quality, which may be different from the previous study. We used the results provided by [36] to perform a re-evaluation of all methods, as shown in Table 6. Meanwhile, visualized images shown in Figure 8 and Figure 9 match well with the quantitative results, which shows PRAGAN’s superior deraining ability and favorable image restoration capability. Note that most other methods used all MIX training sets, while PRAGAN never used all 13,712 images for training, and only 1/4 of the data can achieve the best results.

#### 3.3.2. Real Images

**Figure 10:**
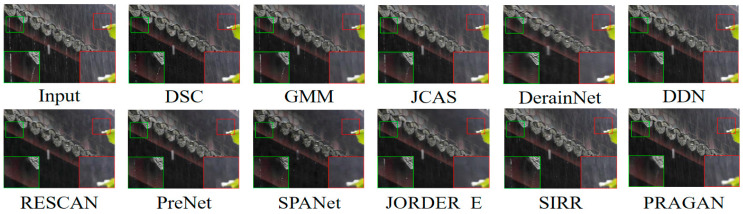
Deraining results on Internet-Data [57] testing set. Best viewed when zoomed in and in color.

Due to the inevitable difference between synthetic rain streaks and real data, this section lists the comparison results of our proposed PRAGAN with other methods on real deraining datasets. According to the results provided by [61], we conducted experiments on two datasets, namely Internet-Data [57] and SPA-Data [62]. For Internet-Data [57], we only provide visual comparison, given that it has no ground truth to allow a quantitative comparison. We pretrained the ViT discriminator for this section with a new dataset that contained Train200 and Internet-Data, for which the mini-batch size was 32, while other configurations were the same as the previous pretraining. In the adversarial training, given that the overall dataset Train200 has only 200 image pairs, we did not use the reselecting data algorithm. PSNR and SSIM comparisons on SPA-Data [62] are shown in Table 7 and a visual comparison on Internet-Data [57] is displayed in Figure 10.

## 4. Conclusions

In this study, we propose a novel generative adversarial network consisting of a pretrained ViT discriminator and a progressive recurrent attention generator for single-image deraining tasks. First of all, we propose a parameter sharing recurrent neural network for image deraining. Secondly, we propose a new pretrained ViT discriminator for image deraining in a GAN. Compared with PatchGAN, ViT in the pretrained stage shows more stable convergence. Finally, we propose a data reselecting algorithm DRA, which can not only make efficient use of training data on small datasets, but also promote the deraining performance of our model on large datasets. We have shown extensive ablation studies and comparative experiments to fully validate the effectiveness of our proposed PRAGAN on both synthetized and real datasets. A more in-depth investigation on image deraining and GAN will be carried out in the future.

## Figures and Tables

**Table 1:** Ablation studies on network structure and loss function. A1 represents the results of removing the CBAM model and A2 shows the predicted value of the last loop as the next input of the network. A3 is the case where MSEloss is used to measure the training effects of three loops. A4 represents the results of removing ConvLSTM. A5 and A6 are the results of adding three losses to the same loop for training and then performing one and three inferences. A7 is the overall network structure with progressive recurrent loss.

	A1	A2	A3	A4	A5	A6	A7
PSNR	24.21	24.31	24.58	24.75	24.77	24.77	24.79
SSIM	0.861	0.869	0.846	0.872	0.871	0.872	0.874

**Table 2:** Ablation studies on pretraining the discriminator or not. ViT discriminator can make the generator perform better in image deraining tasks through pretraining on 128 × 128 image patches.

	Test100 PSNR/SSIM	Rain100H PSNR/SSIM	Rain100L PSNR/SSIM	Test1200 PSNR/SSIM
No pretraining	21.89/0.837	22.99/0.799	24.47/0.861	25.71/0.873
Pretraining	22.55/0.855	23.54/0.813	25.94/0.890	25.95/0.877

**Table 3:** Performance comparison of pretrained ViT discriminator on 256 × 256 image patches. The model was trained on Rain800 [21] and tested on Test100 [21].

	PSNR	SSIM
No pretraining	24.78	0.870
Pretraining	24.87	0.871

**Table 4:** Studies of reselecting data on small-scale training set. The model was trained on Rain800 [21] and tested on Test100 [21]. r represents the proportion of reselected data to the total and 1/4 means fixed quarter of total data.

	r = 20	r = 10	1/4	r = 4
PSNR	23.87	24.83	25.79	26.08
SSIM	0.844	0.866	0.888	0.889

**Table 5:** Studies of reselecting data on a large-scale training set. r represents the proportion of reselected data to the total and 1/4 means fixed quarter of total data.

	Test100 PSNR/SSIM	Rain100H PSNR/SSIM	Rain100L PSNR/SSIM	Test2800 PSNR/SSIM
r = 20	23.97/0.868	25.52/0.838	28.80/0.898	27.76/0.899
r = 10	24.94/0.885	26.69/0.861	30.32/0.929	27.85/0.902
1/4	27.25/0.911	27.09/0.877	31.22/0.933	27.90/0.905
r = 4	27.54/0.912	27.51/0.884	32.77/0.955	27.97/0.906

**Table 6:** Comparative results on synthetic deraining datasets, all models were directly tested on Test1200 [20]. For MPRNet [59], we retrained it with the same number of iterations using the same experimental configuration as our proposed method. Specifically, MPRNet [59] was trained for 63 epochs with all training data.

	Test100 PSNR/SSIM	Rain100H PSNR/SSIM	Rain100L PSNR/SSIM	Test1200 PSNR/SSIM
DerainNet [18]	21.90/0.837	13.67/0.573	26.36/0.873	22.24/0.848
DDC [58]	22.63/0.825	14.51/0.499	26.75/0.858	27.59/0.882
DIDMDN [20]	21.56/0.811	16.31/0.556	23.71/0.804	27.00/0.883
SEMI [57]	21.39/0.781	15.50/0.519	24.05/0.820	24.95/0.841
RESCAN [55]	23.09/0.830	24.86/0.783	27.46/0.864	27.14/0.869
UMRL [56]	23.92/0.883	24.85/0.835	27.73/0.929	29.59/0.922
PreNet [22]	24.03/0.872	25.75/0.861	31.64/0.949	30.86/0.926
MSPFN [36]	26.97/0.898	27.42/0.864	31.66/0.921	31.59/0.928
MPRNet [59]	26.24/0.894	27.73/0.867	32.65/0.951	31.84/0.929
PRAGAN	27.71/0.916	27.41/0.883	32.54/0.957	32.20/0.934

**Table 7:** Comparisons on real-world dataset SPA-Data [61].

Methods	PSNR	SSIM
Input	34.15	0.927
DSC [63]	34.95	0.942
GMM [60]	34.30	0.943
JCAS [64]	34.95	0.945
Clear [5]	32.66	0.942
DDN [58]	34.70	0.934
RESCAN [55]	34.70	0.938
JORDER_E [65]	34.34	0.936
SIRR [57]	34.85	0.936
PRAGAN	34.96	0.951

## Data Availability

All the datasets used for training the model of this paper are from Internet.
